# Supplementary material for: Blood Circulating CD133+ Extracellular Vesicles Predict Clinical Outcomes in Patients with Metastatic Colorectal Cancer
Source: Cancers (Basel). 2022 Mar 7;14(5):1357. doi: 10.3390/cancers14051357 (PMC8909146; doi:10.3390/cancers14051357)
Supplement: Supplementary file 1 [file cancers-14-01357-s001.zip › cancers-1608499-supplementary.pdf]

cancers

MDPI

Blood Circulating CD133+ Extracellular Vesicles Predict Clinical Outcomes in Patients with Metastatic Colorectal Cancer

Davide Brocco , Pasquale Simeone , Davide Buca , Pietro Di Marino , Michele De Tursi , Antonino Grassadonia , Laura De Lellis , Maria Teresa Martino , Serena Veschi , Manuela Iezzi , Simone De Fabritiis , Marco Marchisio , Sebastiano Miscia , Alessandro Cama , Paola Lanuti and Nicola Tinari

Table S1. mCRC patients' characteristics.

Variable	n=54 (%)
Age (%)	
≥65	35 (64.8)
<65	19 (35.2)
Sex (%)	
Male	39 (72.2)
Female	15 (27.8)
ECOG PS	
0	31 (57.4)
1-2	23 (42.6)
Primary Tumor Location	
Right-sided Colon	11 (20.4)
Left-sided Colon	22 (40.7)
Rectum	21 (38.9)
Tumor grading	
1	2 (3.7)
2	38 (74.5)
3	11 (21.6)
Unknown	3 (5.6)
RAS/B-RAF mutations	
K-RAS mutated	22 (40.7)
N-RAS mutated	2 (3.7)
B-RAF mutated	2 (3.7)
Wild type	27 (50.0)
Unknown	1 (1.9)
Number of metastatic sites (%)	
1	25 (46.3)
2	20 (37.0)
≥3	9 (16.7)
Liver Metastasis	
Yes	15 (27.8)
No	39 (72.2)
Lung Metastasis	
Yes	32 (59.3)

No	22 (40.7)
Line of therapy	
1	36 (66.7)
2	13 (24.1)
≥3	5 (9.3)
Systemic Therapy	
Chemotherapy + Cetuximab/Panitumumab	17 (31.5)
Chemotherapy + Bevacizumab	18 (33.3)
Chemotherapy + Aflibercept	2 (3.7)
Chemotherapy	10 (18)
Regorafenib	3 (5.6)
Cetuximab/Panitumumab	4 (7.4)

Table S2. List of flow cytometry specificities and reagents.

Reagent*	Fluorochrome/Reagent	Vendor	Clone	Cat. Number	Volume per test (µl)
Lipophilic Cationic Dye (LCD)	-	BD Biosciences	-	626267	0.5
Phalloidin-FITC	FITC	BD Biosciences	-	626267	0.5
CD133/2	PE	Miltenyi Biotec	293C3	130-113-186	1
EpCAM	PerCP-Cy5.5	BD Biosciences	(EBA-1)	347199	5
CD45	BV510	BD Biosciences	HI30	626266	5

Table S3. Spearman rank correlation coefficients (p-value) between blood circulating EVs and selected clinical-pathological factors in patients with CRC (n=54).

		ECOG PS	Sex	Age	Primary Tumor Location	Tumor Grading[a]	K-RAS mutation	Liver metastasis	Lung metastasis	Number of metastatic sites
**Total EVs**	Correlation Coefficient	0.11	0.06	0.15	-0.25	-0.01	0.05	-0.17	0.13	-0.12
	Sig. (2-tailed)	0.42	0.68	0.28	0.07	0.95	0.71	0.21	0.36	0.38
**CD133+ EVs**	Correlation Coefficient	0.12	0.09	-0.06	-0.09	0.01	0.06	0.03	-0.23	-0.05
	Sig. (2-tailed)	0.38	0.51	0.68	0.53	0.93	0.66	0.86	0.10	0.71
**EPCAM+ EVs**	Correlation Coefficient	0.17	0.03	-0.03	-0.06	-0.13	0.13	-0.06	-0.07	-0.17
	Sig. (2-tailed)	0.22	0.86	0.86	0.67	0.36	0.34	0.69	0.63	0.23

a) Histologic tumor grading was evaluated as recommended by AJCC (8th edition of AJCC-TNM).

Table S4. Comparison of EV concentration in blood samples collected before first line systemic treatment (n=36) and after at least one line of therapy (n=41).

	Treatment Naïve	Post-treatment	p-value
**Median Total EVs/µl (95% CI)**	5530.0 (4431.0-6538.0)	3626.0 (2772.0-5362.0)	0.18
**Median CD133+ EVs/µl (95% CI)**	56.1 (35.0-111.3)	37.2 (14.4-95.0)	0.15
**Median EPCAM EVs/µl (95% CI)**	50.9 (34.2-72.2)	53.2 (28.0-73.5)	0.94

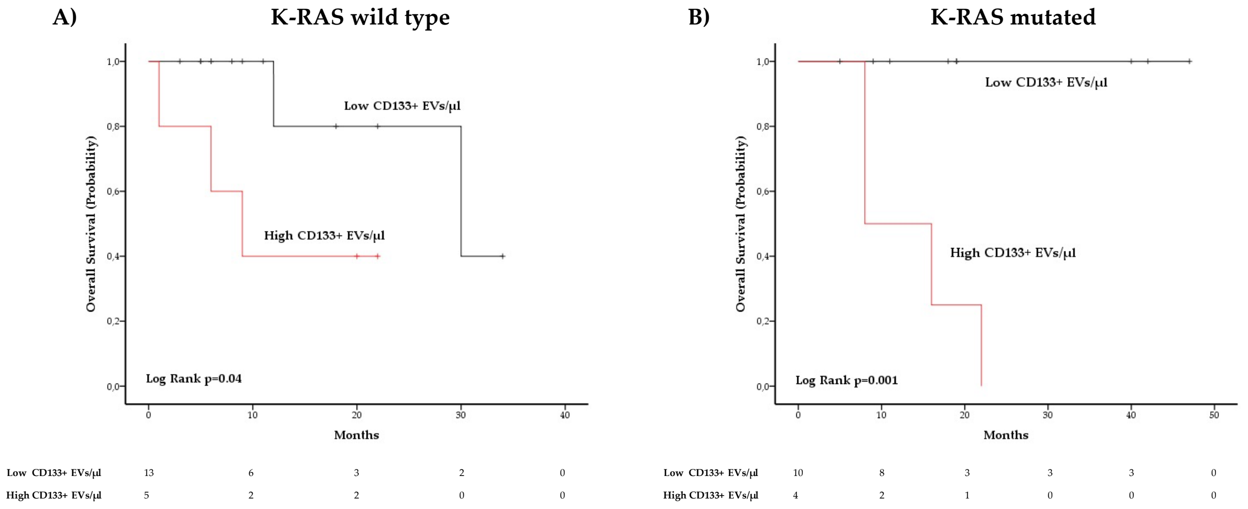

Figure S1. Kaplan-Meier (KM) curves showing the relationship between overall survival and blood concentration of CD133+ EVs in patients with K-RAS wild type **(A)** and K-RAS mutated **(B)** colorectal tumors.

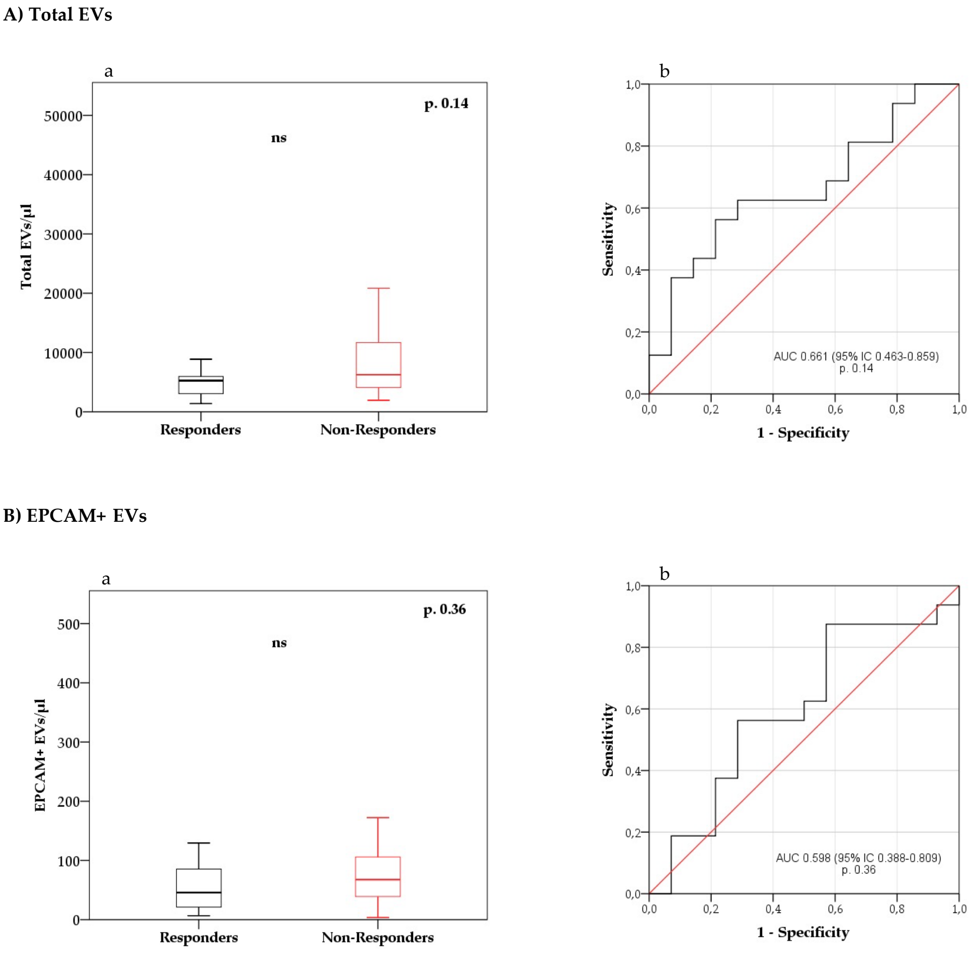

Figure S2. Relationship between treatment response and blood circulating total and EPCAM+ EV concentration at treatment baseline. **Panel A:** (a) Box plot diagram and (b) Receiver operating curve comparing blood concentration of total EVs between responders and non-responders. **Panel B:** (a)

Box plot diagram and (b) Receiver operating curve analyzing difference in blood concentration of EPCAM+ EVs between responders and non-responders.

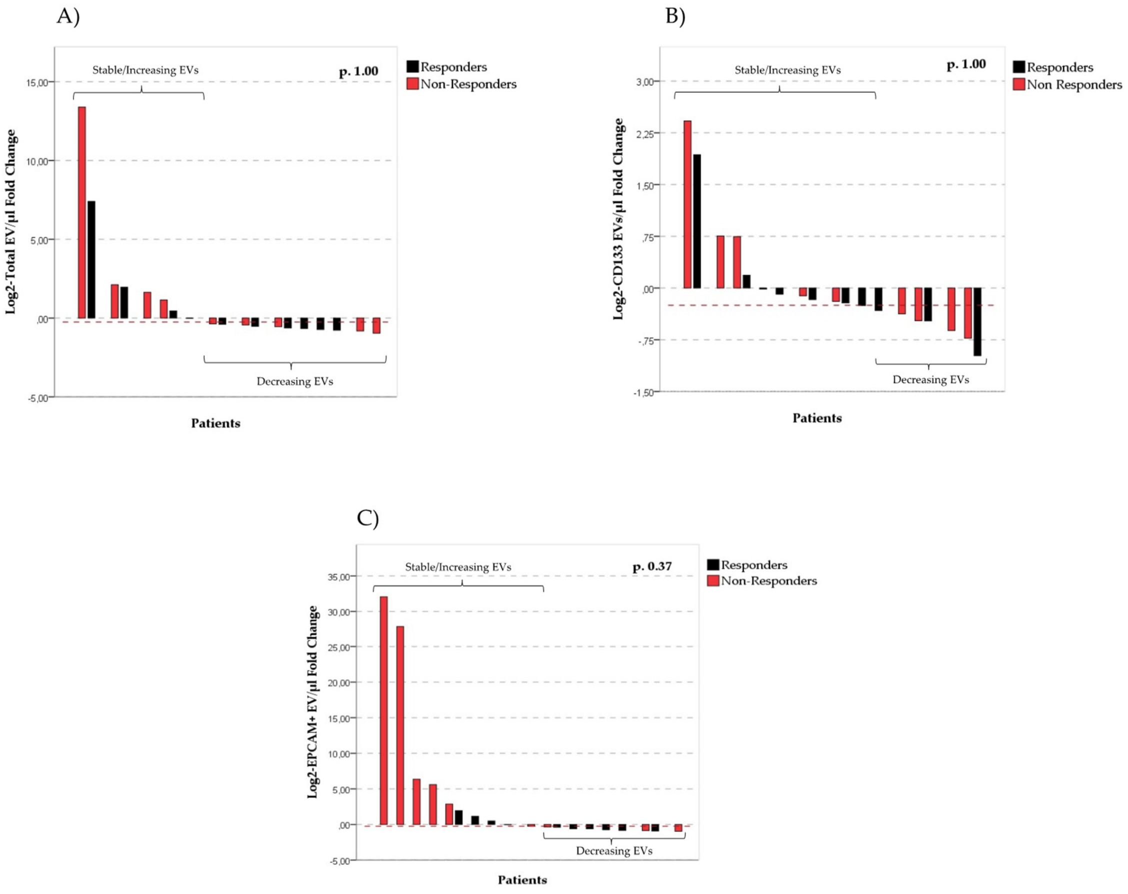

Figure S3. Waterfall plots depicting distributions of responders and non-responders according to variations in blood concentration of total **(A)**, CD133+ **(B)** and EPCAM+ **(C)** EVs during treatment.
